# Inflammation modulates expression of laminin in the central nervous system following ischemic injury

**DOI:** 10.1186/1742-2094-9-159

**Published:** 2012-07-03

**Authors:** Kyungmin Ji, Stella E Tsirka

**Affiliations:** 1 Department of Pharmacology, Stony Brook University, Stony Brook, NY, 11794-8651, USA

**Keywords:** Extracellular matrix proteins, Inflammation, Ischemia, Laminin

## Abstract

**Background:**

Ischemic stroke induces neuronal death in the core of the infarct within a few hours and the secondary damage in the surrounding regions over a long period of time. Reduction of inflammation using pharmacological reagents has become a target of research for the treatment of stroke. Cyclooxygenase 2 (COX-2), a marker of inflammation, is induced during stroke and enhances inflammatory reactions through the release of enzymatic products, such as prostaglandin (PG) E_2_.

**Methods:**

Wild-type (WT) and COX-2 knockout (COX-2KO) mice were subjected to middle cerebral artery occlusion (MCAO). Additionally, brain slices derived from these mice or brain microvascular endothelial cells (BMECs) were exposed to oxygen-glucose deprivation (OGD) conditions. The expression levels of extracellular matrix (ECM) proteins were assessed and correlated with the state of inflammation.

**Results:**

We found that components of the ECM, and specifically laminin, are transiently highly upregulated on endothelial cells after MCAO or OGD. This upregulation is not observed in COX-2KO mice or WT mice treated with COX-2 inhibitor, celecoxib, suggesting that COX-2 is associated with changes in the levels of laminins.

**Conclusions:**

Taken together, we report that transient ECM remodeling takes place early after stroke and suggest that this increase in ECM protein expression may constitute an effort to revascularize and oxygenate the tissue.

## Background

Cerebral ischemia results in neurological disability and constitutes the third leading cause of death in the US. It has devastating consequences as it results in death of neurons at the infarct core within a few hours after the blockade of blood flow to the brain [1]. Although re-establishment of blood flow using fibrinolytics is critical, inflammation that develops following the initial ischemic episode is a major mechanism by which cells in the penumbra degenerate [2], and therefore pharmacological strategies are formulated to limit this delayed phase of damage. Major mediators of inflammatory events are the members of the cyclooxygenase (COX) family. The two major COX isoforms are COX-1 and COX-2 and they catalyze the first step in the transformation of arachidonic acid to prostaglandins (PGs) and thromboxanes. In the central nervous system (CNS), COX-1 and COX-2 are expressed constitutively on neurons, whereas COX-2 is induced on microglia and astrocytes by inflammatory stimuli or injury [3,4]. COX-2 is considered a proinflammatory mediator leading to PG synthesis [5]. Prostaglandin E_2_ (PGE_2_) induces fever and pain, increases vascular permeability and recruit inflammatory cells to injury sites [6]. However, evidence suggests that COX-2 is also involved in inflammation reduction/resolution [7,8]. The COX-2 inhibitors NS398 and indomethacin have differential effects depending on their administration time: early administration during a pleurisy model suppressed inflammation, but late administration exacerbated inflammation through PGE_2_ and 15-deoxy-δ12,14-prostaglandin J_2_ (15d-PGJ_2_) [7]. In a different paradigm, PGE_2_ reduced proinflammatory mediators release from mast cells and inflammatory cells recruitment in lungs [9], suppressed inducible nitric oxide synthase (iNOS) and tumor necrosis factor (TNF)α, but enhanced interleukin 10 and interleukin 13 expression in LPS-stimulated microglia [10,11]. COX-2 also oxygenates anti-inflammatory endocannaboids [12] that protect against ischemic death [13]. These studies suggest that COX-2 can drive or resolve inflammation; therefore, careful regulation of COX-2 may be important for reducing inflammation-mediated neurodegeneration [14,15].

The extracellular matrix (ECM) of the CNS is essential for maintenance of brain homeostasis. Although its exact composition is not defined, it is thought that hyaluronan, tenascin-C, and proteoglycans are present in brain parenchyma [16], and fibronectin (FN) and laminin in the brain vasculature [17,18]. Proteoglycans such as aggrecan, versican, and brevican constitute perineuronal nets majorly [19,20], and tenascin is involved in regulation or promotion of neurite outgrowth [21-23]. Moreover, the structure of these molecules is changed in response to brain injury such as stroke [24,25]. The ECM and secreted neurotrophic or other factors, such as vascular endothelial growth factor (VEGF) or brain-derived neurotropic factor (BDNF), are involved in neuronal reorganization and recovery in MCAO brains [24,26-31]. Among the ECM proteins, laminin is rich primarily in the basement membranes of the endothelial cells of the blood–brain barrier (BBB) [32]. Laminin levels have been reported to increase or decrease during CNS injury [29,33]. Laminins are present in 16 isoforms that are composed of α, β and γ polypeptides. Different laminin isoforms have unique distribution, and are temporally and spatially regulated [34]. Several of the laminin subunits have been shown to be expressed in the rodent brain, including α1 to 5, β1, and γ1 [35,36], and the β3 and γ1 chains have been reported in sprouting neurons and rat astrocytes [37,38]. Laminin α2 is localized in the basal lamina of cerebral blood vessels, and may be important for the selective filtration capability of BBB [39]. The expression of laminin is upregulated in endothelial cells and astrocytes within 24 h following ischemia and stab wounds [40,41].

The work described here investigated whether COX-2 can modulate ECM changes induced by ischemic injury. We focused on laminin as one abundant component of the ECM. Our results indicate that laminin levels on blood vessels are regulated by COX-2 following permanent or transient ischemia. Understanding the functional outcome and timing of laminin expression regulation by COX-2 in the progression of ischemia-induced neuronal damage could suggest a basis for potentially rationalizing drug specific interference with ischemia.

## Methods

### Animals and *in vivo* experiments

All animal procedures were approved by the Stony Brook University Institutional Animal Care and Use Committee (IACUC). Adult wild-type (C57BL6; WT) mice were obtained from Jackson Laboratory (Bar Harbor, ME, USA). Cyclooxygenase knockout mice (COX-2KO in the C57Bl6 background) were provided by Dr SK Dey (Cincinnati Children's Hospital). Mice were bred in house at Stony Brook. For middle cerebral artery occlusion (MCAO), mice were anesthetized and underwent permanent MCAO (pMCAO) using a heat-blunted, small 6–0 siliconized monofilament (Ethicon, Somerville, NJ, USA). A fiberoptic probe was glued to the parietal bone (2 mm posterior and 5 mm lateral to bregma) and connected to a laser-Doppler flowmeter (Periflux System 5010, Perimed, Stockholm, Sweden) for continuous monitoring of cerebral blood flow in the ischemic territory center. Celecoxib (Biovision, Milpitas, CA, USA) was given at 5 mg/kg intraperitoneally (in 50 % dimethylsulfoxide (DMSO)) 30 minutes before the injury. The animals were killed at different times. The infarct area was visualized by cresyl violet and 2,3,5-triphenyltetrazolium chloride (TTC) staining.

### Tissue preparation

Mice were anesthetized after surgery and perfused with saline solution, followed by 4 % paraformaldehyde (PFA) in 0.1 M phosphate buffer, pH 7.2, for tissue fixation. Brains were obtained and post fixed overnight at 4 °C in 4 % PFA. Fixed brains were stored at 4 °C in 30 % sucrose solution until they sank. Six separate series of 20 μm coronal brain sections were obtained with a cryostat. For protein preparation, mice were anesthetized and perfused with saline. Brains were sliced with Mice Brain Slicer Matrix (ASI Instruments, Warren, MI, USA) and a razor blade. The slice including the ipsilateral sides (ischemic lesion) was selected, and tissue blocks (1.0 × 1.0 × 1.0 mm^3^) in the lesion of ipsilateral sides and in the same area of contralateral (not ischemic) sides were collected, and stored at −70 °C until use.

### Measurement of Infarct volume

To quantify the infarct volume TTC staining was used: mice were killed and perfused with saline after MCAO. The brain slices, obtained as described above, (2 mm) were incubated for 15 minutes in 2 % TTC (Sigma-Aldrich, St. Louis, MO, USA) at 37 °C, and fixed in 4 % PFA at 4 °C. TTC stains viable brain tissue dark red, whereas infarcted tissue areas remain unstained (white). To measure the TTC-negative area, serial sections from each animal were viewed in a Nikon E600 microscope, photographed and the area measured using NIS-Elements software (ImageJ). The infarct volume was calculated as sum of (area × section thickness) for each animal.

### Oxygen-glucose deprivation (OGD)

Immortalized human brain microvascular endothelial cells (BMECs) were a gift from Dr M Stins at Johns Hopkins University, School of Medicine [42]. BMECs were cultured in RPMI1640 medium, supplemented with 10 % NuSerum, 10 % fetal bovine serum (FBS), minimal essential medium (MEM) vitamins, MEM non-essential amino acids, 1 mM sodium pyruvate, 2 mM d-glutamine, 30 μg/ml endothelial growth supplement, 5 U/ml heparin, and penicillin/streptomycin at 37 °C in 5 % CO_2_. The cells form a monolayer connected via tight junctions that can form and model an *in vitro* blood–brain barrier [43]. For *in vitro* ischemia, the cells were maintained in glucose-free and serum-free (OGD conditioned) medium under 1 % O_2_/5 % CO_2_ at 37 °C (Oxycycler C4, Biospherix, Redfield, NY, USA) for 3 days. Afterwards, the cells were removed from the hypoxic chamber and replaced with pre-OGD conditioned medium in a humidified aerobic incubator at 37 °C for 4 h recovery [44].

### Immunoblotting, immunohistochemistry, and immunofluorescence

For immunoblotting, cells or tissue from the ipsilateral or contralateral hemisphere were lysed in 50 mM Tris–HCl (pH 7.4) containing 1 % Nonidet P-40, 0.25 % Na-deoxycholate, 150 mM NaCl, and protease inhibitors cocktail (Sigma-Aldrich) using a homogenizer on ice, incubated for 30 minutes, and centrifuged. The extracts were run on a reducing 10 % sodium dodecylsulfate polyacrylamide gel electrophoresis (SDS-PAGE) and transferred to polyvinylidene fluoride (PVDF) membrane (Immobilon-P; Millipore, Billerica, MA, USA). The blots were incubated using primary antibodies (Table 1) overnight at 4 °C; followed by incubation with horseradish peroxidase-labeled secondary antibody (Invitrogen, Carlsbad, CA, USA) for 1 h at room temperature, and enhanced chemiluminescence (ECL) (Pierce Chemical Co., Rockford, IL, USA). After stripping, the membranes were reblotted with mouse anti-α-tubulin (Upstate Biotechnology, Lake Placid, NY, USA) antibody. Expression levels were quantified using the ImageJ software, normalized against α-tubulin. For immunohistochemistry and immunofluorescence, sections were fixed in 4 % PFA in phosphate-buffered saline (PBS) for 30 minutes. After washing in PBS, they were blocked in 0.2 % Triton X-100 and 1 % bovine serum albumin (BSA) in PBS. The primary antibodies used were listed in Table 1. The sections were incubated with primary antibody in 0.2 % Triton X-100 and 1 % BSA in PBS at 4 °C overnight. After rinsing in PBS, the sections were incubated with biotinylated secondary antibodies, the avidin/biotin system, and visualized using 3,3'-diaminobenzidine, or Alexa Fluor488-conjugated or Alexa Fluor555-conjugated secondary antibodies (Invitrogen) for 1 h. The sections were rinsed in PBS, coverslipped, and examined using confocal microscopy or epifluorescence microscopy.

**Table 1:** Antibodies used for immunostaining or immunoblotting

**Antigen**	**Antibody**	**Dilution**	**Source**	**Catalog no.**	**Method**
NeuN	Mouse monoclonal	1:1,000	Chemicon	MAB377	IHC
Iba-1	Rabbit polyclonal	1:1,000	Wako	019-19741	IHC
TLR9	Rabbit polyclonal	1:500	Imgenex	IMG431	WB
CD14	Goat polyclonal	1:1,000	Santa Cruz Biotechnology	sc-6999	WB
Fibrin	Mouse monoclonal	1:200	A gift of Dr Galanakis	[45]	WB
Collagen IV	Rabbit polyclonal	1:500	Chemicon	AB756	WB
Pan-laminin	Rabbit polyclonal	1:1,000	Sigma-Aldrich	L9393	IHC
		1:5,000			WB
α-Tubulin	Mouse monoclonal	1:7,000	Upstate Biotechnology	05-829	WB
Occludin	Mouse monoclonal	1:250	Zymed	33-1500	IHC
HIF-1α	Rabbit polyclonal	1:1,000	Bethyl Laboratories	A300-286A	WB
COX-2	Mouse monoclonal	1:500	BD Biosciences	610203	WB
Mac-2	Rat monoclonal	1:1,000	Cedarlane	CL8942AP	WB
CD45	Rat monoclonal	1:500	BD Biosciences	553076	IHC
VEGF	Rabbit polyclonal	1:200	Thermo scientific	RB-9031	WB
GFAP	Rabbit polyclonal	1:1,000	DAKO	Z0334	IHC

COX-2, cyclooxygenase 2; GFAP, glial fibrillary acidic protein; HIF-1α, hypoxia-inducible factor 1α; Iba-1, ionized calcium binding adaptor molecule 1; IHC, immunohistochemisty; TLR9, Toll-like receptor 9; VEGF, vascular endothelial growth factor; WB, western blot.

### Reverse transcription polymerase chain reaction (RT-PCR)

Total RNA was extracted using TRIzol (Invitrogen) and cDNA was prepared using reverse transcriptase, according to manufacturer’s instructions (Invitrogen). The PCR primers used are listed in Table 2. PCR products were separated by electrophoresis in 1 % agarose gels and detected under ultraviolet (UV) light.

**Table 2:** Primer sequences for reverse transcription polymerase chain reaction (RT-PCR)

**Gene**	**Forward/reverse**	**Sequences (5’-3’)**
Laminin α2	F	GCCGCACTCCTGGACCAACC
	R	TGCCAGTGCTCGCAGCCATC
Laminin α4	F	ATCGAGGGGAGCGCAGTGGT
	R	GCAGAACCGGGGTGTGCCTC
Laminin α5	F	CTGTGGAGCCCGCCTGTGTG
	R	CCCCACGCGACACTGGTCAC
Laminin β1	F	CGAACCTGCAGCGAGTGCCA
	R	GTTCCGCTGCTGGGCTCTGG
Laminin β3	F	CCAGCGCACACGGCTTCTCA
	R	GTGCCCTGCCGAAGGTTCCC
Laminin γ1	F	CTGTGAGACTGTGCCGCCCG
	R	ACAGTGCTGGCCGGTGATGC
Laminin γ2	F	CGGCTGTGTGTAGCGGGGTG
	R	CAGCAGGAGCGCGACACCAA
actin	F	GCTCGTCGTCGACAACGGCT
	R	CAAACATGATCTGGGTCATCTTCTC

Using permanent MCAO we followed the effect of ischemia and local inflammation, and using transient MCAO we followed the effect of systemic inflammatory cell infiltration on changes in ECM proteins during post-injury CNS reorganization.

### Statistics

Statistical significance was assessed by analysis of variance (ANOVA), followed by Student-Newman-Keuls multiple comparison tests. All analyses were performed using SPSS, V.8.0 (SPSS, Chicago, IL, USA). Values are means ± SEMs of at least three independent experiments using at least five animals per experimental procedure and mouse strain, unless otherwise indicated.

## Results

### Laminin expression is transiently upregulated in wild-type, but not COX-2KO, mice after MCAO

**Figure 1:**
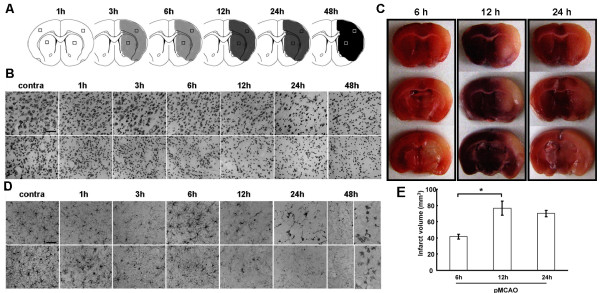
**Neuronal death and Iba**^**+**^**microglia in ischemic brain following middle cerebral artery occlusion (MCAO).** Sections at the indicated times after MCAO were stained with cresyl violet (**B**), 2,3,5-triphenyltetrazolium chloride (TTC) (**C**), and Iba-1 antibody (**D**). Contralateral sides were used as controls. Upper and lower sections are from the cortex and striatum, respectively. The sections were stained with cresyl violet; the areas of neuronal death were revealed by the absence of cresyl violet stain and are represented in the cartoons as gray shading (**A**). (**E**) Infarct volume was measured as described in the Methods section. Data were obtained from five animals per timepoint. Values are means ± SEMs of at least three independent experiments of each group unless otherwise indicated. Bars, 50 μm.

We used permanent MCAO (pMCAO) to investigate how ischemia-induced local inflammatory events affect the ECM. The extent of neuronal death and local inflammation (microglial cell activation) were assessed over time. The number of Nissl bodies was reduced at 3 h compared to that in contralateral sides following pMCAO (Figure 1A,B and Additional file 1: Figure S1) and the infarct volume was increased (Figure 1E). Regions not stained with TTC indicating cerebral infarction were observed within 6 h following pMCAO (Figure 1C). The state of microglial activation was also visualized using the Iba-1 marker, which is specific for macrophages/microglia (Figure 1D). Change of microglial morphology to an ameboid state was observed using Iba-1 staining within 1 to 3 h after pMCAO, and sustained until at least 48 h after the injury. Similarly, the infiltration of leukocytes was assessed after MCAO (Additional file 1: Figure S1): immunoreactivity for CD45 was evident 1 h after MCAO, as well as at later timepoints, namely at 24 and 48 h.

**Figure 2:**
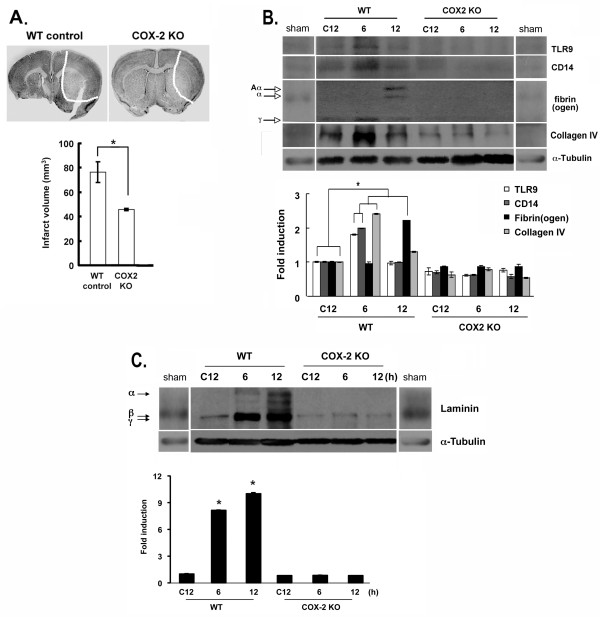
**Impaired laminin expression in COX-2 knockout (COX-2KO) mice following ischemic injury.** Lower magnification images of cresyl violet staining ((**A**), upper) on brain sections from wild-type (WT control) and COX-2KO, mice that were subjected to middle cerebral artery occlusion (MCAO). Sections shown were collected 12 h after MCAO and stained with cresyl violet. White dotted lines demarcate the infarct. *P <0.05 compared to WT control or WT vehicles. (**B**,**C**) Extracellular matrix (ECM) protein expression in extracts from ipsilateral sides after MCAO was analyzed for Toll-like receptor 9 (TLR9), CD14, fibrin(ogen), collagen IV (B), and laminin levels (α, β and γ subunits) (C) and was quantified using the ImageJ software, normalized against α-tubulin and graphed as fold difference in mean intensity. *P <0.01 compared to the contralateral WT and COX-2KO sides at 12 h (C12). Data were obtained from at least five animals in each group.

As COX-2 is one of the critical contributors to microglial activation [4], we assessed the extent of neuronal death and other parameters in COX-2^−/−^ mice (COX-2KO) relative to wild-type (WT) animals. COX-2KO mice were subjected to MCAO alongside WT animals. At 12 h after injury the extent of neurodegeneration in COX-2KO mice was decreased compared to WT mice (Figure 2A, upper and infarct volume graph), as reported previously [46]. Microglial activation was similarly reduced (data not shown). When we evaluated the expression of common ECM components, we found that proteins such as laminin, fibrinogen, fibronectin (not shown), and collagen IV were upregulated in ischemic brain (Figure 2B,C). The markers tested exhibited a transient increase after MCAO in WT animals. This transient increase was not detectable in the sham and contralateral (not ischemic) side, marked in Figure 2B, C as C12 (contralateral side 12 h post MCAO). In COX-2KO tissue extracts, the transient increase was not observed at the time points examined. This result suggests that the MCAO injury probably induces and COX-2 could be involved in an inflammation-mediated change in the ECM protein expression.

Since laminin was the ECM protein whose levels were predominantly affected by MCAO, and given the known involvement of laminin in revascularization, we examined whether the MCAO and ECM changes also involved changes in the expression of VEGF, a factor known for mediating angiogenesis and neoangiogenesis. As shown in Additional file 2: Figure S2, the levels of VEGF were elevated in wild-type animals at 6 and 12 h post MCAO (peak at 6 h), but this increase was drastically attenuated in COX-2KO mice, suggesting that COX-2 could be involved in laminin-mediated angiogenesis [47].

### Laminin is increased in endothelial cells after ischemic injury

**Figure 3:**
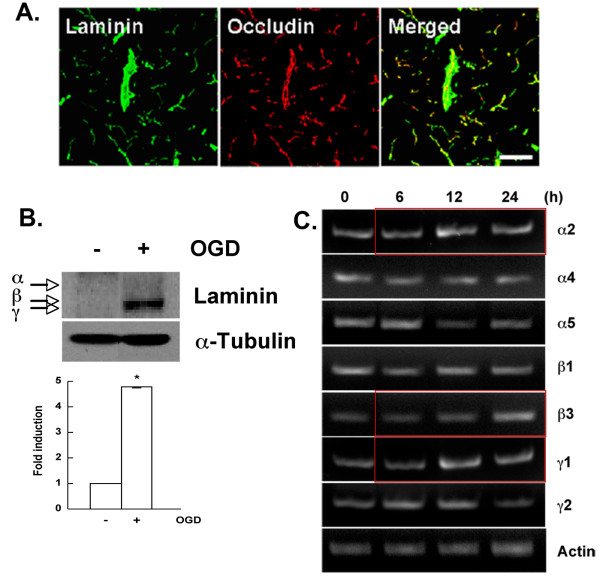
**Laminin expression increased on blood vessels following transient middle cerebral artery occlusion (tMCAO).** (**A**) At 6 h post MCAO, sections were stained with antibodies against anti-pan-laminin and occludin. Data are representative of results from at least five animals. Scale bar, 100 μm. (**B**) Brain microvascular endothelial cells (BMECs) were challenged with oxygen-glucose deprivation (OGD) and assayed for laminin expression. *P <0.01 compared to control. (**C**) RT-PCR for laminin subtypes at indicated times after MCAO.

Among the ECM markers investigated, the most prominent change in protein levels was observed in laminin, as shown in Figure 2C. In the subsequent experiments we focused our investigation on laminin and its expression after stroke. To ascertain the cellular localization of laminin, we used occludin as an indicator of endothelial cells on the blood vessels since laminin has been considered as a major component of the ECM of the BBB [17]. Laminin expression was prevalent on occludin^+^ cells (Figure 3A), demonstrating that endothelial cells were one cellular source of upregulated laminin [36,48]. To further confirm that laminin expression was increased on endothelial cells, brain microvascular endothelial cells (BMECs) were subjected to conditions of OGD, as described in the Methods section, and analyzed by immunoblotting. Laminin expression, in particular β and γ subunits, was upregulated on endothelial cells following injury (Figure 3B).

The antibody used in Figures 2C and 3A,B was a pan-laminin antibody, so we sought to determine which laminin subunits are upregulated after MCAO. We used semiquantitative RT-PCR for each laminin subunit in extracts from the ipsilateral side of MCAO animals at the indicated times (Figure 3C). The analysis showed that laminins α2, β3, and γ1 were upregulated within 24 h (Figure 3C).

### Pharmacological inhibitors of COX-2 modulate laminin levels after MCAO

**Figure 4:**
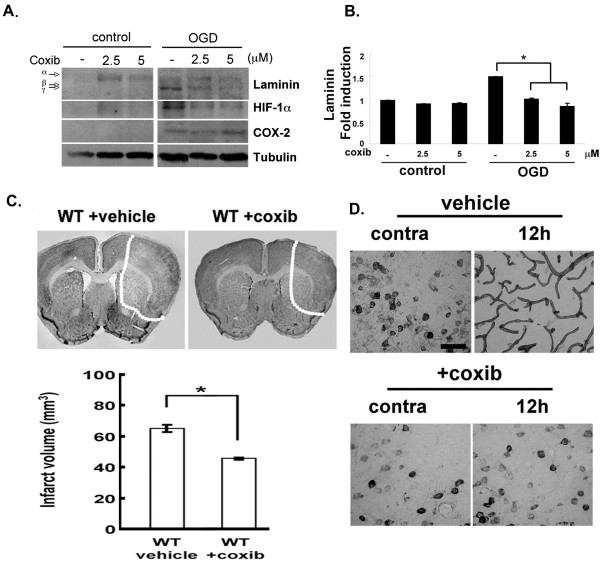
**Impaired laminin expression in cyclooxygenase 2 (COX-2)-inhibited cells and mice following ischemic injury.** (**A**) Brain microvascular endothelial cells (BMECs) were treated with celecoxib (coxib, 2.5 and 5 μM) and subjected to oxygen-glucose deprivation (OGD). (**B**) Laminin expression was determined in cell extracts. α-Tubulin normalized protein loading. Bands were quantified by densitometry and plotted as mean intensity. The value of laminin expression in bar graph was averaged over all experiments for the expression of all laminin subunits; *P <0.01. (**C**) Lower magnification images of cresyl violet staining (upper) of brain sections from wild-type (WT) mice treated with vehicle alone (WT + vehicle) and the COX-2 inhibitor coxib (WT + coxib) prior to middle cerebral artery occlusion (MCAO). White dotted lines demarcate the infarct. Infarct volume was measured. *P <0.05 compared to WT vehicle (lower). (**D**) Sections shown were collected 12 h after MCAO and stained with pan-laminin antibodies. Data are representative of results from three independent experiments. Bars, 50 μm.

COX-2 deficiency modulated the levels of several ECM markers following MCAO (Figure 2B,C). Focusing again on laminin, we assessed whether this effect of COX-2 was acute or rather a potential developmental effect. To address this concern, we impaired COX-2 activity pharmacologically using celecoxib (coxib). We used first OGD in culture, as described above. Coxib was given at 2.5 and 5 μM during the OGD and the levels of laminin were examined using immunoblotting. As shown in Figure 4A,B, the OGD samples had higher levels of laminin compared to control samples. When coxib was used, the levels of laminin were decreased down to control levels. Similar to laminin, the hypoxia inducible factor 1α (HIF-1α), which a classic marker upregulated during ischemia or OGD [49], was decreased in the presence of coxib. The levels of α-tubulin or COX-2 remained the same in all conditions. Coxib was also administered *in vivo* to wild-type mice immediately before MCAO. More intact Nissl bodies were observed in coxib-treated mice (WT + coxib) compared to control ones that had not been treated with coxib (WT + vehicle) (Figure 4C). In these Nissl^-^ area, laminin expression was dramatically decreased in coxib-treated mice (+coxib) compared to control mice (vehicle) (Figure 4C,D) and was reminiscent of laminin expression levels in COX-2KO mice. Notably, the intense staining indicating increased expression of blood vessels (endothelial cells) was not observed in the coxib-treated animal tissues. These results suggest that COX-2 can regulate acutely laminin expression.

### COX-2 acts through E-prostanoid 3 (EP3) receptors to modulate ECM protein expression

**Figure 5:**
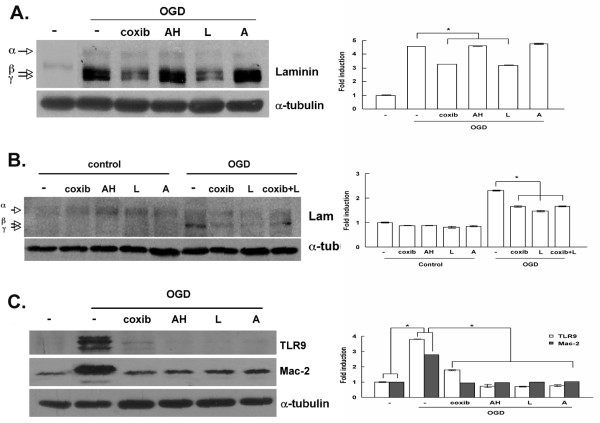
**Decreased laminin expression in cyclooxygenase 2 (COX-2)-inhibited endothelial cells following oxygen-glucose deprivation (OGD).** (**A**) Cells were pretreated with antagonists, subjected to OGD and western blot analysis. AH, AH6809 (1 μM; Ki = 350 nM), EP2 antagonist; L, L798,106 (1 nM; Ki = 0.3 nM), EP3 antagonist; A, AH23848 (1 μM; IC_50_ = 0.26 μM), EP4 antagonist. (**B**) Laminin expression in COX-2-inhibited endothelial cells following OGD. Cells were pretreated with antagonists, subjected to OGD and western blot analysis. (**C**) Brain microvascular endothelial cells (BMECs) were pretreated with celecoxib (coxib) and antagonists for 15 minutes, then subjected to OGD and western blot analysis using antibodies against Toll-like receptor 9 (TLR9) and Mac-2. α-Tubulin was used to normalize protein loading. Quantification was performed using the ImageJ software, normalized against α-tubulin. *P <0.01 compared to control. Data are representative of results from at leas five experiments.

We assessed whether the effect of COX-2 on laminin and ECM protein expression was mediated through prostaglandins and prostaglandin receptors. We used pharmacological inhibitors of EP receptors on BMECs and subjected them to OGD. Of the antagonists used, SC51089 (EP1), AH6809 (EP2), L798,106 (EP3) and AH23848 (EP4), only the EP3 antagonist decreased laminin levels (Figure 5A). The combination of coxib and L798,106 did not result in further laminin decrease expression (Figure 5B). This result suggests that COX-2 regulates laminin expression via EP3 receptor activation, which mainly function to inhibit adenylyl cyclase via Gi activation [50].

As discussed earlier, in Figure 2, other ECM protein components were also increased after MCAO, and their expression correlated with increased inflammation. We evaluated the expression of markers not directly associated with the COX-2 pathway (TLR9, Mac-2, CD14) in the presence of coxib or EP antagonists, and found that their expression was also decreased when the COX-2 pathway was inhibited (Figure 5C). These results suggested that laminin and other ECM protein expression increase is associated with brain inflammation.

**Figure 6:**
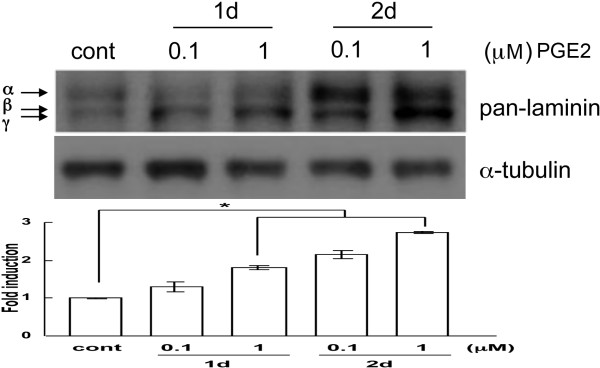
**Laminin protein levels increase after treatment of endothelial cells with prostaglandin E**_**2**_**(PGE**_**2**_**).** BMECs were treated with 0.1 and 1 μM PGE_2_ for 1 or 2 days. At the end of the incubation period the cells were lysed and subjected to western blot analysis to reveal laminin protein levels. α-Tubulin was used to normalize protein loading. Data are representative of three experiments. The α, β and γ subunits are indicated by arrows. *P <0.01 compared to control.

Because the EP3 inhibitor inhibited the induction of laminin (Figure 5A), we explored whether incubation of BMECs with PGE_2_ would result in increase of laminin expression. The cells were treated with increasing concentrations of PGE_2_ over a period of 2 days. As shown in Figure 6, a strong upregulation of all laminin subunits was evident at the 1-day but mostly at the 2-day timepoint. This result further supports the involvement of COX-2/PGE_2_ in the processes that lead to laminin induction.

## Discussion

ECM components play regulatory roles in various cellular events [51-53], including healing/repair processes after injury [54]. For some ECM proteins it has been reported that their expression increases in ischemia and stab wounds [40,41], but it is not known what its contribution to the injury outcome is. Focusing on one ECM component, we report that the increase in laminin depends on COX-2 activity.

Laminin upregulation has been linked to wound healing stimulation [54,55] and angiogenesis. In a skin wound model, application of laminin peptides increased the wound coverage and repair and was accompanied by enhanced angiogenesis. Laminins α2, β3, and γ1 are increased in brain following transient ischemic injury. These laminins have been reported to be expressed in brain [33,38,56]. The biological roles of laminin subtypes and trimer molecules are largely unknown, thus, information about their functions derives from the phenotype of deficient or knockout mice. Laminin α1 is involved in epiblast differentiation [34]. Laminin α2 is expressed in basal lamina surrounding neurons and muscle [57]. α2 deficiency causes congenital muscular dystrophy [58]. Laminin β1 deficient mice lack basement membranes and display defects in the neuromuscular synapse [34]. Laminin β3 is expressed on the basal lamina and ECM of all cerebral microvessels [59]. The γ1 subunit is rapidly degraded during excitotoxicity [60] or ethanol-induced neurodegeneration [61]. Although these subunits were upregulated after MCAO, it is not clear if they form a single laminin isoform or distinct heterotrimers. Moreover, it is not clear if they would act through the same receptors, integrins, dystroglycan or syndecan [34].

COX-2 activity enhances the production of VEGF [47], which in turn stimulates angiogenesis. The downstream effectors of COX-2 would be different prostaglandins such as PGI_2_, PGD_2_, PGE_2_, PGF_2α_, and thromboxane (Tx). These prostaglandins bind to the receptor with the greatest affinity: I prostanoid (IP) receptor binds PGI_2_, DP binds PGD2, EP binds PGE_2_, FP binds PGF_2α_, and TP binds TxA_2_, and they are basically G protein-coupled prostanoid receptors (GPCR) [62,63]. Among the prostaglandins, prostaglandin E_2_ (PGE_2_) is the most widely produced prostaglandin in the body, and considered to participate in inflammation associated with redness, swelling and pain [64,65] and has significant effects on proliferation, the apoptosis of lymphocytes and the regulation of cytokine production in T cells [64]. PGE_2_ has the most known receptors with EP subtypes characterized as EP1 to EP4. Depending on the model of CNS injury, different EP receptors mediate the cellular signals. In a model of pulmonary emphysema angiogenesis was mediated by an EP2 agonist [66], whereas bone marrow cells expressing EP3 receptor enhanced angiogenesis during chronic inflammatory conditions [67]. In Lewis lung carcinoma the proangiogenic microenvironment was regulated via COX-2/EP3 or EP4 signaling [56]. In our study the effects of COX-2 on laminin expression were mediated through EP3 receptors. Our results suggest that although ECM changes are associated with the general acute inflammatory processes after MCAO, they may result from specific signaling changes. This latter hypothesis is supported by the fact that not all EP antagonists affect laminin expression, yet all affect other marker expression.

The observation that eventually laminin expression was recovered in COX-2-deficient animals at 2 days (data not shown) suggests that COX-2 deficiency delays the upregulation of the ECM protein laminin, potentially due to the release of different prostaglandins at early and late inflammatory stages and/or neuronal recovery during ischemic injury.

If the initial response of the ischemic tissue is an effort to promote neoangiogenesis to re-establish the blood flow [68,69], our data suggest that the early inflammatory reactions may be aiding such a process. Our results also point to molecular components critical for increased vascularization, suggesting that inhibition of molecules, such as EP3, may not be beneficial for stroke outcome.

## Conclusions

Our study shows that following MCAO a transient upregulation of proteins of the ECM is observed, primarily of proteins associated with the endothelial cells of the CNS blood vessels. The presence of COX-2 and the pathway it initiates are important for this upregulation. Although we do not currently know why this ECM protein changes occur, we speculate that they constitute an early endogenous proangiogenic attempt from the tissue to re-establish oxygenation of the surrounding tissue.

## Competing interests

The authors declare no competing interests.

## Authors' contributions

KJ carried out the experiments described, analyzed data and drafted the manuscript. SET initiated and designed the study, analyzed data, and critically reviewed the manuscript. Both authors read and approved the final manuscript.

## Supplementary Material

Additional file 1 **Figure S1.** Leukocyte infiltration during middle cerebral artery occlusion (MCAO). Sections at the indicated times after MCAO were stained with anti-CD45 antibody to visualize the timing of the infiltration of leukocytes into the central nervous system (CNS) parenchyma following MCAO. Click here for file

Additional file 2 **Figure S2.** Vascular epithelial growth factor (VEGF) expression following middle cerebral artery occlusion (MCAO). VEGF protein expression was analyzed in 6 and 12 h extracts from ipsilateral sides after MCAO and was compared to levels on the contralateral side. α-Tubulin was used to normalize protein loading. *P <0.01 compared to control. Click here for file
