# Supplementary figures and images for: Inflammation modulates expression of laminin in the central nervous system following ischemic injury
Source: J Neuroinflammation. 2012 Jul 3;9:159. doi: 10.1186/1742-2094-9-159 (PMC3414761; doi:10.1186/1742-2094-9-159)

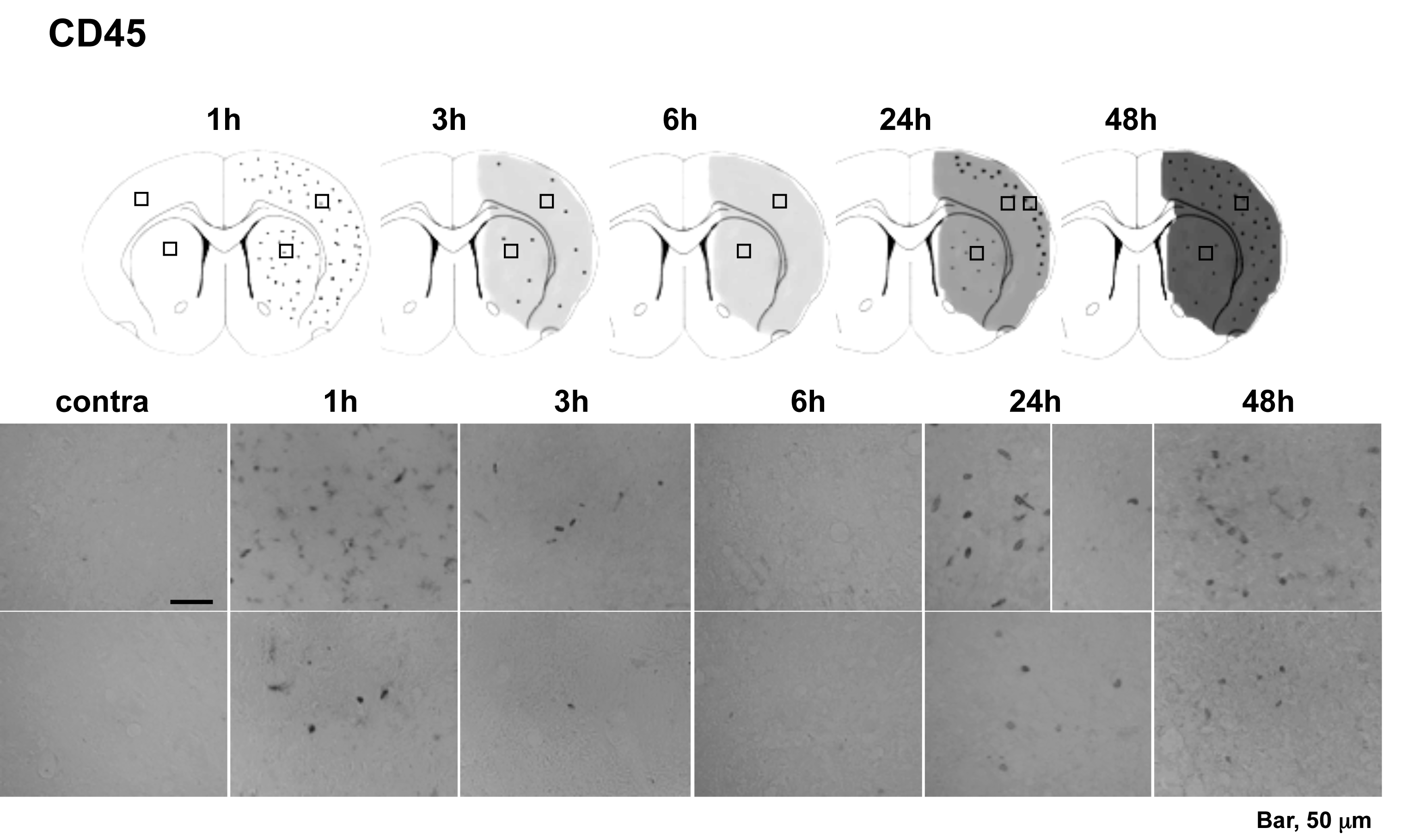

Supplement: Additional file 1 — Figure S1. Leukocyte infiltration during middle cerebral artery occlusion (MCAO). Sections at the indicated times after MCAO were stained with anti-CD45 antibody to visualize the timing of the infiltration of leukocytes into the central nervous system (CNS) parenchyma following MCAO. [file 1742-2094-9-159-S1.tiff]

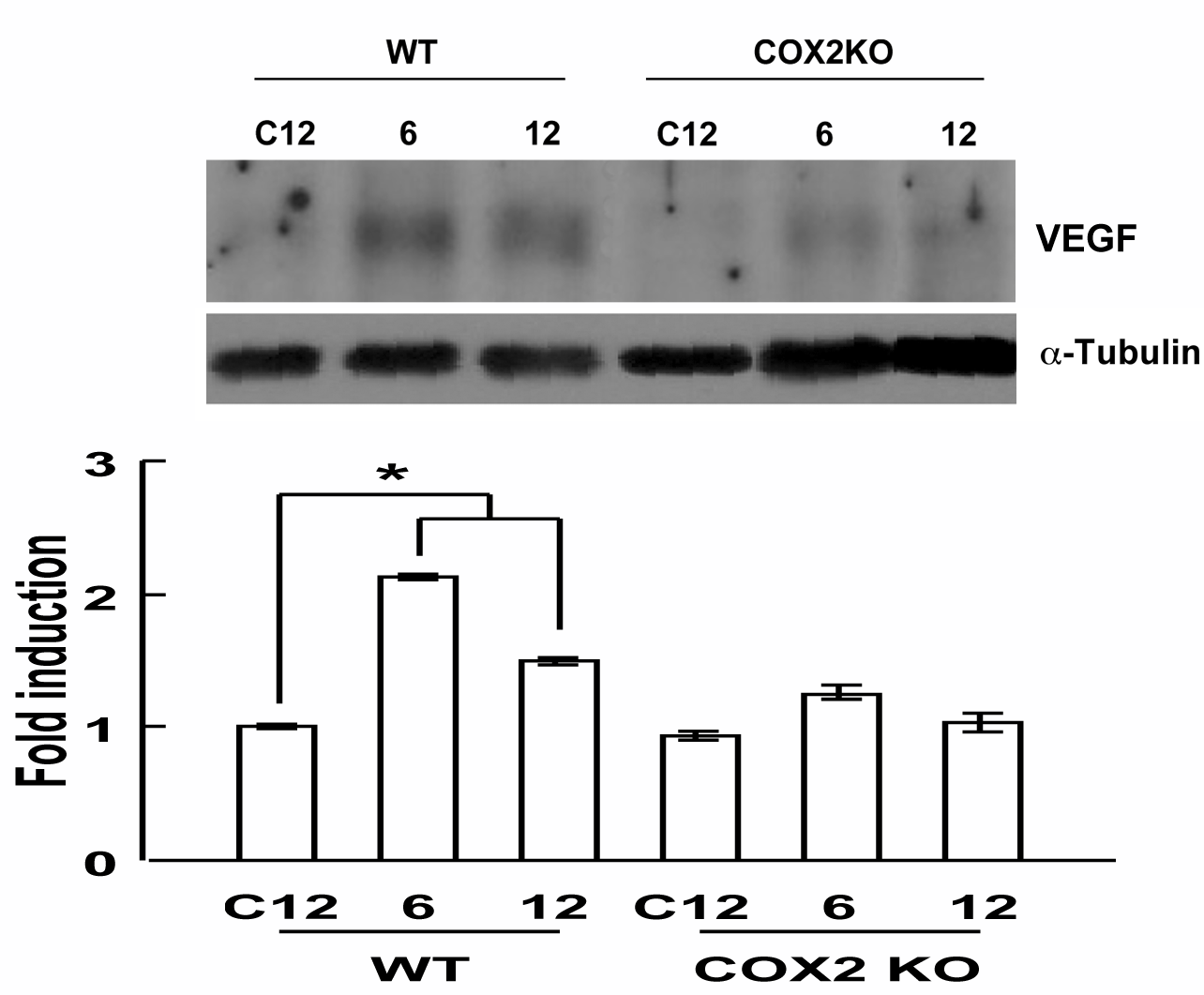

Supplement: Additional file 2 — Figure S2. Vascular epithelial growth factor (VEGF) expression following middle cerebral artery occlusion (MCAO). VEGF protein expression was analyzed in 6 and 12 h extracts from ipsilateral sides after MCAO and was compared to levels on the contralateral side. α-Tubulin was used to normalize protein loading. *P <0.01 compared to control. [file 1742-2094-9-159-S2.tiff]
